# Auditory- and Vestibular-Evoked Potentials Correlate with Motor and Non-Motor Features of Parkinson’s Disease

**DOI:** 10.3389/fneur.2017.00055

**Published:** 2017-02-27

**Authors:** Ali Soliman Shalash, Dalia Mohamed Hassan, Hanan Hani Elrassas, Mohamed Mosaad Salama, Edna Méndez-Hernández, José M. Salas-Pacheco, Oscar Arias-Carrión

**Affiliations:** ^1^Department of Neurology, Ain Shams University, Cairo, Egypt; ^2^Audiology Unit, Department of Otorhinolaryngology, Ain Shams University, Cairo, Egypt; ^3^Institute of Psychiatry, Ain Shams University, Cairo, Egypt; ^4^Department of Toxicology, Mansoura University, Mansoura, Egypt; ^5^Instituto de Investigación Científica, Universidad Juárez del Estado de Durango, Durango, Mexico; ^6^Unidad de Trastornos del Movimiento y Sueño, Hospital General Dr. Manuel Gea González, Ciudad de México, Mexico

**Keywords:** Parkinson’s disease, motor, non-motor, vestibular, auditory, evoked potentials

## Abstract

Degeneration of several brainstem nuclei has been long related to motor and non-motor symptoms (NMSs) of Parkinson’s disease (PD). Nevertheless, due to technical issues, there are only a few studies that correlate that association. Brainstem auditory-evoked potential (BAEP) and vestibular-evoked myogenic potential (VEMP) responses represent a valuable tool for brainstem assessment. Here, we investigated the abnormalities of BAEPs, ocular VEMPs (oVEMPs), and cervical VEMPs (cVEMPs) in patients with PD and its correlation to the motor and NMSs. Fifteen patients diagnosed as idiopathic PD were evaluated by Unified Parkinson’s Disease Rating Scale and its subscores, Hoehn and Yahr scale, Schwab and England scale, and Non-Motor Symptoms Scale. PD patients underwent pure-tone, speech audiometry, tympanometry, BAEP, oVEMPs, and cVEMPs, and compared to 15 age-matched control subjects. PD subjects showed abnormal BAEP wave morphology, prolonged absolute latencies of wave V and I–V interpeak latencies. Absent responses were the marked abnormality seen in oVEMP. Prolonged latencies with reduced amplitudes were seen in cVEMP responses. Rigidity and bradykinesia were correlated to the BAEP and cVEMP responses contralateral to the clinically more affected side. Contralateral and ipsilateral cVEMPs were significantly correlated to sleep (*p* = 0.03 and 0.001), perception (*p* = 0.03), memory/cognition (*p* = 0.025), and urinary scores (*p* = 0.03). The oVEMP responses showed significant correlations to cardiovascular (*p* = 0.01) and sexual dysfunctions (*p* = 0.013). PD is associated with BAEP and VEMP abnormalities that are correlated to the motor and some non-motor clinical characteristics. These abnormalities could be considered as potential electrophysiological biomarkers for brainstem dysfunction and its associated motor and non-motor features.

## Introduction

Parkinson’s disease (PD) is a common neurodegenerative disorder caused by degeneration of midbrain dopaminergic neurons of substantia nigra (SN) producing its primary motor cardinal features (1). In addition to the motor symptoms, PD patients develop a variety of non-motor symptoms (NMSs), which significantly impair their quality of life. The NMSs consist of autonomic dysfunction, sensory symptoms, neuropsychiatric disturbances, sleep disorders, fatigue, and gastrointestinal disorders (2). Those NMSs are associated with dopaminergic and non-dopaminergic dysfunctions including serotoninergic, noradrenergic, and cholinergic systems (3, 4). Degeneration of several brainstem nuclei and their connections is responsible—at least partially—for different neurotransmitters disruption, resulting in different NMSs (3, 5).

Lewy bodies (LB) and Lewy neurites (LN) composed of alpha-synuclein are the pathological hallmarks of PD (6, 7). Alpha-synuclein pathology related to NMS was described in brain hemispheres, brainstem, spinal cord, and peripheral nervous system (5). Recent studies emphasized the importance of brainstem as the habitat of degeneration of nuclei responsible for different NMSs (8). Moreover, some NMS-related pathological changes might have specific distribution and anatomical localization in various levels of the brainstem. For example, depression and REM sleep behavior disorder are related to degeneration of pontine nuclei (5).

These reports are consistent with Braak’s proposal of pathological progression of PD, which starts caudally from the dorsal motor vagal nucleus in the medulla and then ascends in the brainstem and finally involves neocortex (9, 10). Thus, most brainstem nuclei are involved in early stages (I–III) that explain the preclinical and early emergence of NMS, while SN is involved in stage III (3, 5). Recently, Seidel et al. demonstrated the widespread of LB and LN in brainstem nuclei and fiber tracts including vestibular nuclei (4). Therefore, diagnostic tools exploring disruption of lower brainstem nuclei and related NMS are needed for early diagnosis of PD (11).

Brainstem auditory-evoked potential (BAEP) and vestibular-evoked myogenic potential (VEMP) responses represent a valuable tool for brainstem assessment as the neural pathways of both ocular VEMPs (oVEMPs) and cervical VEMPs (cVEMPs) pass through the brainstem (12). VEMPs are short latency manifestations of vestibulo-ocular reflex connecting VIII and III cranial nuclei and vestibulo-collic reflex connecting VIII and XI cranial nuclei that originate from the utricle and saccule, respectively (12). Previous studies described impaired BAEP and VEMP responses in PD patient compared to controls that were attributed to underlying brainstem dysfunction (13–16). Furthermore, impairment of these responses was related topographically to other brainstem lesions. Principally, the BAEP and oVEMP responses are affected in upper brainstem (midbrain) lesions, while cVEMP responses are involved in the lower brainstem (pontine and upper medullary) lesions (17–19). Therefore, they have a localizing value of brainstem dysfunction at different levels.

In the current study, we hypothesized that NMSs of PD related to brainstem dysfunction could be related to changes in VEMPs and BAEP. Accordingly, the aim of this study was to explore the abnormalities of BAEPs and VEMPs in patients with PD and its correlation to the motor and NMSs of PD.

## Materials and Methods

### Materials

Fifteen patients diagnosed as idiopathic PD and 15 age-matched control subjects were included in the current prospective study. PD patients were recruited from the movement disorders outpatient clinic at Ain Shams University Hospitals, Cairo, Egypt in the period between 2013 and 2015. Recruited patients were diagnosed as idiopathic PD according to the British Parkinson’s Disease Society Brain Bank criteria (20). Exclusion criteria included dementia (MMSE score <24), improper neck movements that interfere with audiological assessment, middle ear diseases, and hearing thresholds exceeding 50 dBnHL.

All subjects were evaluated using Unified Parkinson’s Disease Rating Scale (UPDRS), Hoehn and Yahr scale (H&Y), and Schwab and England scale (S&E) in “medication off” and “on” states by a movement disorders expert. Different UPDRS subscales were estimated including the activity of daily living (UPDRS I), a motor (UPDRS III), UPDRS I, and total UPDRS scores. Furthermore, main motor symptoms subscores were calculated such as tremor (items 20 and 21 of UPDRS), rigidity (item 22), bradykinesia (items 18, 19, 23, and 24), axial signs (items 27, 28, 29, and 30), postural instability/gait disability score (items 13, 14, 15, 29, and 30) (21). The NMSs were measured for all patients by the Non-Motor Symptoms Scale (NMSS) administrated by the movement disorders expert (2). Control subjects were age- and sex-matched normal volunteers and provided a reference of auditory and vestibular work up. Informed consent was taken from all subjects before participation in the present study. The study protocol was approved by the Ethics Committee of Faculty of Medicine, Ain Shams University. All subjects gave written informed consent by the Declaration of Helsinki.

### Procedures

#### Audio-Vestibular Works Up

Basic audiological evaluation to assess the peripheral auditory system, pure-tone (PTA), and speech audiometry was done using the two-channel audiometer Grason-Stadler Inc. (GSI, Eden Prairie, MN, USA) model 61 calibrated according to ANSI (1969) in a sound-treated room IAC model 1602 (IAC Acoustics, UK). The middle ear functions were tested through the acoustic immittance meter Grason-Stadler Inc. (GSI, Eden Prairie, MN, USA) model 33. BAEP and VEMP were done to all subjects of the study using the ICS Chartr EP 200—GN Otometrics (Denmark)-evoked potential system.

For the BAEP assessment, the active electrode was mounted to the middle of the forehead “Fpz,” the reference electrode to the ipsilateral mastoid “M1,” and the ground to the contralateral one “M2.” The test procedures followed Sininger protocol (22). Analysis of BAEP was done quantitatively to assess the absolute latencies of waves I, III, and V and interpeak latencies of these waves (I–III, III–V, and I–V). This was done both at high stimulus level “90 dBnHL” and at lower intensities down to thresholds. The interaural latency difference and the latency/rate function were studied at high stimulus intensity. Qualitative analysis of the waveform morphology comprised the subjective judgment on the shape and the quality of the waveforms.

### Vestibular-Evoked Myogenic Potentials

#### oVEMP Test

Monaural stimulation with contralateral eye recording was employed for recording oVEMPs (23). Three surface electromyography (EMG) electrodes were placed on the face just inferior and at the center of lower eyelid (the active electrode), the chin (the reference electrode), and the forehead (the ground electrode). During recording, all subjects were instructed to look upward at a small fixed target >2 m from the eyes, while the vertical eye position was at an angle of approximately 30–35° above horizontal. The oVEMP response included the initial negative–positive biphasic waveform comprised peaks n1 and p1. Two runs were performed for each test to confirm the reproducibility of results. The latencies of peaks n1 and p1, amplitude n1–p1, and interaural amplitude difference (IAD) ratio were measured.

#### cVEMPs Test

The subject was seated with the head rotating sideways toward one shoulder to activate the sternocleidomastoid (SCM) muscle. The active electrode was placed at midpoints of each SCM muscle on symmetrical sites, the reference electrode on the suprasternal notch, and the ground electrode on the forehead. Monaural acoustic stimulation with ipsilateral recording was employed for recording cVEMPs. The n13–p13 wave latencies, amplitudes, and IAD were measured (24).

**Figure 1:**
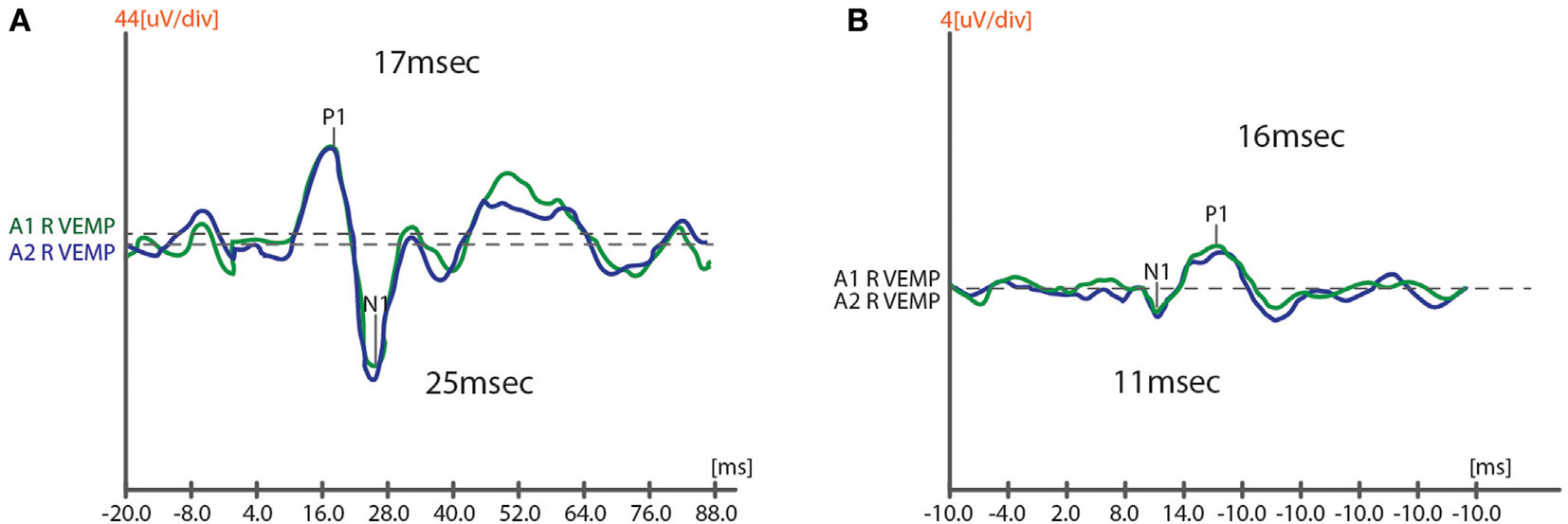
**A sample of recorded response of cVEMP (A) and oVEMP (B) in one of study subjects**. cVEMPs, cervical vestibular-evoked myogenic potentials; oVEMP, ocular VEMPs.

Assessment of auditory and vestibular responses was done during medications “On” states to decrease EMG artifacts and ensure patients cooperation. The findings of BAEPs, cVEMPs, and oVEMPs of PD patients were grouped to ipsilateral and contralateral to the clinically more affected (CMA) side and compared to the mean of both sides of the control subjects, then VEMP responses are correlated to different UPDRS Off-scores and NMS scores. Figure 1 shows samples of cVEMP and oVEMP responses of one of the recruited subjects.

### Statistical Analysis

Statistical analyses were performed using the SPSS version 18. Qualitative data were described using number and percent, while quantitative data were described using mean and SD. Association between categorical variables was tested using chi-square test. Comparison between two independent variables was done using independent *t*-test. Correlations between quantitative variables were assessed using Spearman coefficient. The level of statistical significance (*p* value) was set at 0.05 (significant) and 0.01 (highly significant).

## Results

Fifteen patients with idiopathic PD (12 males and 3 females) completed the clinical and audiological assessments. Their mean age was 59.20 ± 10.08 years (ranged from 35 to 70 years), and the duration of illness was 5.50 ± 2.96 years (2–10 years). The mean total UPDRS and mean disease disability (S&E scale) scores were 41.33 ± 30.20 and 68.67 ± 22.30, respectively. Patients were of variable disease stages ranged from stage 2 to 5 of H&Y scale in “Off” state with mean 2.73 ± 0.84. All patients had at least impaired domain of NMS. Sleep/fatigue (86.7%) and mood/cognition (73.3%) were the most frequent reported NMS. Detailed motor and non-motor scores of the PD patients are presented in Tables 1 and 2.

**Table 1:** **Demographic and clinical characters of Parkinson’s disease patients**.

Clinical feature	Mean ± SD (range)
Age (years)	59.20 ± 10.08 (35–76)
Duration of disease (years)	5.50 ± 2.96 (2–10)
Age of onset (years)	53.77 ± 11.49 (25–70)
H&Y off	2.73 ± 0.84 (2–5)
H&Y on	0.93 ± 0.59 (0–2)
S&E off	68.67 ± 22.30 (50–90)
S&E on	90.0 ± 9.26 (70–100)
UPDRS I off	3.33 ± 2.13 (0–6)
UPDRS II off	12.73 ± 7.49 (0–27)
UPDRS III off	30.20 ± 17.49 (2–69)
UPDRS IV	0.67 ± 1.40 (0–4)
UPDRS-total	41.33 ± 30.20 (2–109)
Postural instability/gait disability off	5.20 ± 4.06 (0–16)
Axial off	5.27 ± 4.28 (0–15)

*H&Y, Hoehn and Yahr scale; S&E, Schwab and England scale; UPDRS, Unified Parkinson’s Disease Rating Scale*.

**Table 2:** **Severity and prevalence of non-motor manifestations of Parkinson’s disease patients**.

NMS	Mean ± SD (range)	Prevalence (%)
NMS CVS	0.93 ± 1.67 (0–6)	40
NMS sleep/fatigue	7.07 ± 6.36 (0–22)	86.7
NMS mood and cognition	11.60 ± 12.54 (0–42)	73.3
NMS perception/hallucinations	0.80 ± 1.27 (0–4)	33.3
NMS memory	5.00 ± 6.07 (0–20)	60
NMS GIT	2.80 ± 3.78 (0–9)	53.3
NMS urinary	5.87 ± 8.96 (0–33)	60
NMS sexual	3.93 ± 5.81 (0–16)	46.7
NMS miscellaneous	3.33 ± 4.67 (0–17)	60
NMS total	41.33 ± 30.20 (2–109)	100

*NMS, non-motor symptoms; CVS, cardiovascular system; GIT, gastrointestinal tract*.

### Audiological Work Up

The PD patients had significantly higher PTA thresholds mainly in the high frequencies 4 and 8 kHz bilaterally compared to the age-matched control group (*p* < 0.03). Seven PD subjects (46.7%) had sensorineural hearing loss of mild to moderate degree (bilateral symmetrical in five and unilateral in two).

#### Brainstem Auditory-Evoked Potentials

The ipsilateral and contralateral absolute latencies of wave V (*p* = 0.04) and I–V interpeak (*p* = 0.025 and 0.03) latencies were significantly prolonged compared to controls. Moreover, the wave III and the interpeak I–III latencies were also significantly prolonged (*p* = 0.03 and 0.036, respectively) ipsilateral to the CMA side. Eight patients (53%) had an abnormal BAEP wave morphology.

#### Ocular VEMPs

Absent oVEMP responses were the commonest abnormality and were detected in 47% of PD subjects (*n* = 7). Compared to control group, the latencies of n1 and p1 were significantly prolonged when contralateral to the CMA side (*p* = 0.04 and 0.025), and n1–p1 amplitude was significantly reduced bilaterally (*p* < 0.001).

#### Cervical VEMPs

The cVEMP responses were absent in three patients (20%). Compared to controls, the ipsi- and contralateral p13, and contralateral n23 latencies were significantly prolonged (*p* = 0.04, 0.001, and 0.04, respectively) and bilateral p13–n23 amplitudes were significantly decreased (*p* < 0.001).

### Correlations between BAEP, VEMPs, and UPDRS Scores

The contralateral absolute latencies of waves III and V were significantly correlated to disease severity (H&Y scale) (*r* = 0.610, *p* = 0.028 and *r* = 0.530, *p* = 0.043, respectively) and wave V with rigidity “Off” score (*r* = 0.540, *p* = 0.039). Furthermore, the absence of BAEP waves contralateral to the CMA was significantly correlated to S&E, UPDRS III, and rigidity “Off” scores (*r* = 0.665, *p* = 0.007; *r* = −0.540, *p* = 0.037; and *r* = −0.770, *p* = 0.001, respectively).

The abnormal cVEMP responses contralateral to the CMA side showed significant correlation to “H&Y” disease stage (wave latency) (*r* = 0.689, *p* = 0.013), UPDRS III (*r* = 0.523, *p* = 0.045), rigidity (wave latency) (*r* = 0.634, *p* = 0.027), and bradykinesia “Off” scores (wave absence) (*r* = 0.571, *p* = 0.026). The ipsilateral p13 and n23 wave latencies were also correlated to dyskinesia scores (*r* = 709, *p* = 0.01 and *r* = 634, *p* = 0.027, respectively). Furthermore, the oVEMP responses ipsilateral to the CMA side showed moderate correlation with a trend to significance with UPDRS III, rigidity, and axial “off” scores (*p* = 0.046, 0.05, and 0.049, respectively).

On the other hand, the UPDRS II, tremor, and S&E subscales showed no significant correlations with BAEP and VEMP responses. The duration of the disease, age, and age of onset showed no correlation either with BAEP, cVEMP, and oVEMP responses.

### Correlations between BAEP, VEMPs, and Non-Motor Scores

The BAEP showed minor associations to some non-motor functions scores. The contralateral I–III and I–V interpeak latencies were correlated to NMS-gastrointestinal tract (GIT) scores (*r* = 0.625, *p* = 0.03 and *r* = 0.595, *p* = 0.041, respectively), while the ipsilateral I–III interpeak latency was correlated to sleep/fatigue scores (*r* = 0.586 and *p* = 0.035).

**Figure 2:**
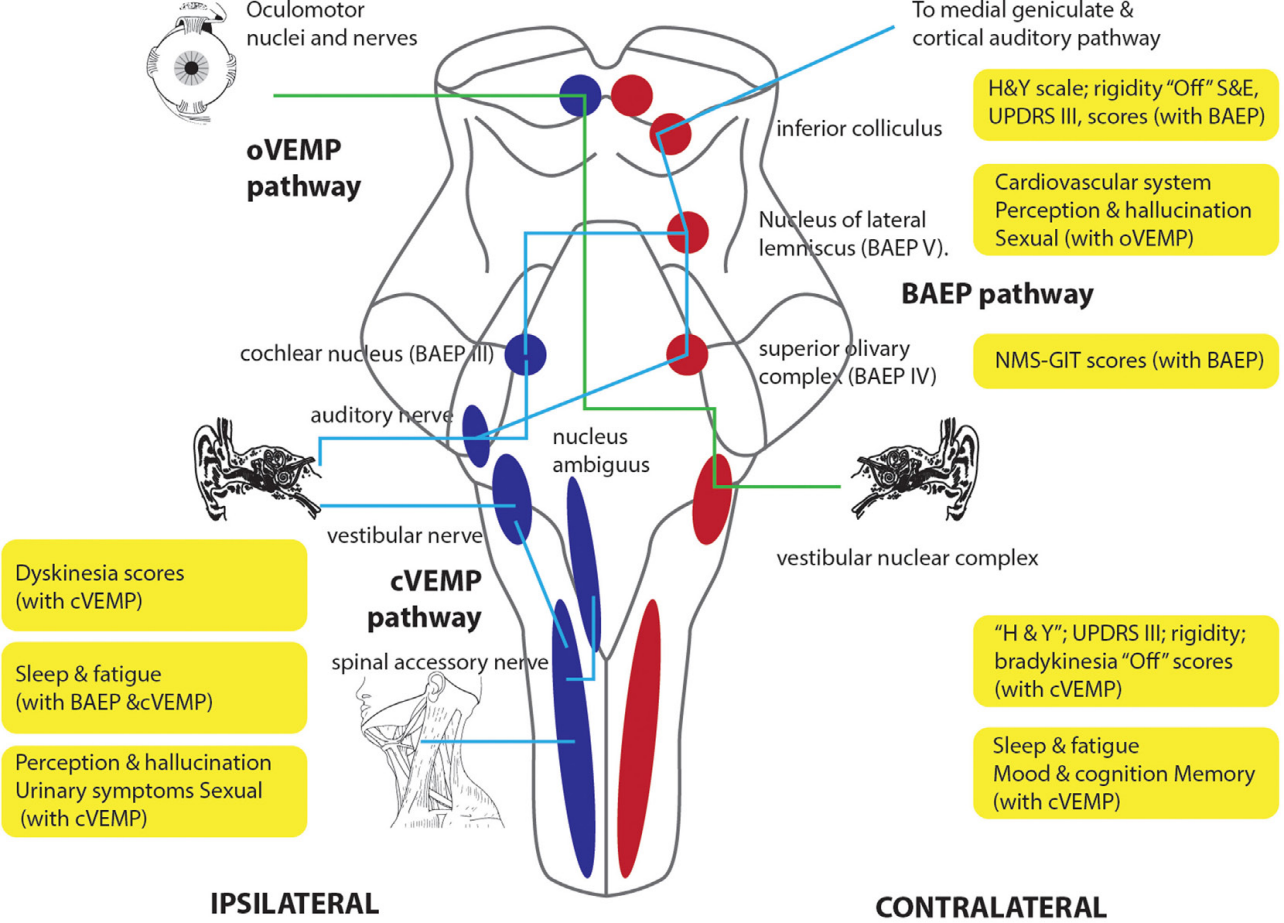
**The schematic diagram describes the topographic distribution of different motor and non-motor manifestations of Parkinson’s disease patients according to its correlation with BAEP, cVEMP, and oVEMP abnormal responses in the current study**. BAEPs, brainstem auditory-evoked potentials; cVEMPs, cervical vestibular-evoked myogenic potentials; oVEMP, ocular VEMPs.

Contralateral and ipsilateral cVEMP responses showed significant correlations to most of NMSS domains. They were significantly correlated to mood/cognition (0.024), sleep/fatigue (*p* = 0.03 and 0.001), perception (*p* = 0.03), memory (*p* = 0.025), and urinary scores (*p* = 0.03). Sexual dysfunction was moderately correlated to the absence of ipsilateral responses (*p* = 0.045). The oVEMP responses showed significant correlations to fewer NMS domains including NMS cardiovascular system (*p* = 0.01), sexual dysfunction (*p* = 0.013), and perception (moderate correlation, *p* = 0.047) (see Table 3; Figure 2). GIT domain showed no significant correlation with VEMP responses.

**Table 3:** **Positive correlation between NMS scores and VEMP responses**.

NMS	Related ARB and vestibular-evoked myogenic potential (VEMP) responses	*r*	*p*
CVS	Contralat ocular VEMPs (oVEMP) absent response	−0.643	0.01
Sleep/fatigue	Ipsilat I–III	0.586	0.035
	Contralat cervical VEMPs (cVEMPs) n23 latency	0.845	0.001
	Contralat cVEMP p13 latency	0.583	0.047
	Ipsilat cVEMP pn amplitude	−0.625	0.03
GIT	Contralat I–III latency	0.625	0.03
	Contralat I–V latency	0.595	0.041
Mood/cognition	Contralat cVEMP n23 latency	0.643	0.024
Perception/hallucination	Ipsilat cVEMP pn amplitude	−0.625	0.03
	Contralat oVEMP p1 latency	0.674	0.047
Memory	Contralat cVEMP pn amplitude	−0.639	0.025
Urinary	Ipsilat cVEMP n23 latency	0.622	0.031
Sexual	Contralat oVEMP n1 latency	0.782	0.013
	Ipsilat cVEMP absent response	0.524	0.045

*NMS, non-motor symptoms; CVS, cardiovascular system; GIT, gastrointestinal tract; Ipsilat, ipsilateral; contralat, contralateral (to clinically more affected side)*.

## Discussion

The current study investigated comprehensively the brainstem dysfunction of PD patients and its relation to motor and NMSs using BAEPs and VEMPs. It confirmed the impairment of BAEP and VEMP responses in patients with PD compared to controls that are related to clinical asymmetry of PD and its cardinal motor features. The BAEPs and cVEMPs were correlated significantly to contralateral motor UPDRS, rigidity, and bradykinesia severity, and ipsilateral dyskinesia scores. Moreover, cVEMP responses were correlated to some non-motor features including sleep/fatigue, mood/cognition, perception, and memory. BAEP and oVEMP responses showed less correlation to the motor and non-motor features. Consequently, it reflects brainstem pathology among PD patients at different levels and highlights the asymmetry of these changes.

In this study, we separated the vestibular responses to ipsilateral and contralateral responses according to CMA side. This approach could reflect the expected asymmetrical brainstem pathology of PD (25). Previous studies confirmed the asymmetry of cardinal motor symptoms that often persists through the course of the disease. This clinical asymmetry is related to asymmetrical degeneration of dopaminergic neurons of SN, striatal dopaminergic receptors, and their cortical connections (8, 25). Furthermore, the clinical asymmetry could be related to NMSs including pain, fatigue, depression, and cognitive dysfunction. This could be explained by asymmetrical underlying pathological changes and the asymmetrical dopaminergic deficiency that contributes to some non-motor features along with other neurotransmitters (8, 25).

The high-frequency hearing impairment associated with PD was detected in the current work similar to prior studies (26–28). Impaired hearing in PD was attributed to peripheral auditory dysfunction (27, 28) and abnormal central auditory processing (29, 30). Likewise, abnormal BAEPs among PD patients were demonstrated inconsistent to previous studies, denoting brainstem auditory conduction delay (26, 31, 32).

Additionally, both the oVEMPs and cVEMPs were affected in the studied PD subjects. The findings of the present study agreed with de Natale et al. who reported that the frequency of alteration of VEMPs in PD patients was 83.3% when considering the combined set of cVEMP, oVEMP, and masseter VEMP responses, with absence being the prevalent alteration in PD (14). Similarly, Pollak et al. showed unilaterally absent VEMP responses in 20 (37%) of PD patients and bilaterally absent responses in 4 patients (7.4%) (33). Lower amplitudes of cVEMPs among PD patients were reported by another study (34). However, a recent study by Pötter-Nerger et al. reported abnormal oVEMPs in mild to moderate PD patients with preserved cVEMPs that were attributed to methodological differences (16). These findings emphasize the extensive brainstem dysfunctions at different anatomical levels (35).

Auditory and vestibular dysfunctions in PD could be explained by different mechanisms. Modulatory effect of dopamine on the excitability of vestibular nuclei is one of these mechanisms (34). Disrupted interconnections of vestibular nuclei with degenerated other brainstem nuclei by PD pathology especially dorsal raphe nuclei is another mechanism (36, 37). Furthermore, direct disruption of vestibular nuclei by PD pathological changes produces these vestibular abnormalities as recently reported (4).

Remarkably, the current study defined correlation of the main motor features of PD with BAEPs and cVEMPs rather than oVEMPs that might be explained by the midbrain and pontine pathological changes and non-involvement of vestibulo-ocular pathways in the pathophysiology of these features. Rigidity and bradykinesia were related to BAEP and cVEMP responses, while tremor was not. The correlations were mainly to the responses contralateral to CMA side, which is consistent with asymmetric nature of PD pathology in SN and their connections (25).

Tremor has different pathophysiology compared to rigidity and bradykinesia and characterized by involvement of the cerebellum–thalamocortical circuit in its pathogenesis (37). This explains the lack of correlation with auditory and vestibular responses. Moreover, recent animal studies demonstrated that brainstem structures such as pontine nuclei and locus coeruleus are involved in the pathophysiology of levodopa-induced dyskinesia (LID) (38). This could explain the correlation seen in the present study between LID and cVEMP wave latencies.

Although the correlation of BAEPs, oVEMPs, and cVEMPs were mainly to one side, yet no differences existed in all tests between the two sides. The medication state of the subjects could be the explanation. All PD subjects underwent the tests during “medication on state” that masked the abnormalities between the two sides as recently reported by Pötter-Nerger et al. (34).

In contrast, previous studies reported a lack of correlation with clinical motor scores (14, 33, 34). Nonetheless, de Natal et al. defined the progression of VEMP abnormalities with increased stage of the disease (14). They used the mean values of VEMP responses on both sides, not about the CMA side, thus underestimating the potential asymmetry that could ameliorate abnormalities. Moreover, differences in experimental conditions during testing, age differences between cases and controls, and different clinical characteristics of recruited patients could explain the inconsistency of results of different studies that addressed the vestibular functions in PD.

Few prior studies investigated the correlation of auditory and vestibular responses with individual NMSs of PD (14, 33). In the current study, sleep and mood domains’ severity demonstrated correlation with cVEMP responses that could be related to associated dysfunction of different pontine nuclei such as locus coeruleus, raphe nucleus, and pedunculopontine nucleus (3). Previous studies reported localizing function of cVEMP responses of pontine lesions associated with different other diseases (17–19). Recently, de Natale et al. found a direct correlation between VEMP changes and REM sleep behavior disorder and postural instability (14). The same study reported a lack of correlation with depression (14), while a study by Pollack et al. reported a correlation of cVEMP with depression and antidepressants use (33).

Unsurprisingly, GIT domain that includes hypersalivation, dysphagia, and constipation had minor correlations. This could be attributed to the high contribution of peripheral pathological changes in GIT and related nerve supply (alpha-synucleinopathy) along with brainstem changes (39–41). Furthermore, GIT symptoms are attributed to dysfunction of the dorsal motor nucleus of the vagus, nucleus ambiguous, and nucleus of a solitary tract located in the medulla (3) that is poorly localized by VEMP responses (13). Correlation of GIT symptoms’ severity to contralateral BAEP latency could be explained by associated advanced disease (42). Similarly, the contribution of sacral spinal cord alpha-synuclein deposition to urinary symptoms explains limited correlation to vestibular responses (43). Likewise, correlation of other non-motor features poorly linked to brainstem dysfunction such as cardiovascular symptoms, attention/memory, and perception/hallucination could be explained by associated disease severity (42). Cognitive deficits of PD are correlated to higher order auditory processing in subcortical–cortical pathways evaluated by event-related potentials (P300) (29, 44).

The current study has different limitations. These limitations include a small number of recruited sample, including patients with different disease severity and stages, and the use of NMSS subscores that describe different NMSs for correlations with vestibular responses. Thus, further, more specific tools for each NMS are required for correlation with the electrophysiological assessment of the larger number of PD patients.

In conclusion, the current study confirms the auditory and vestibular abnormalities among PD patients that reflect brainstem pathological changes. It also correlates these abnormalities to some motor and non-motor features of the disease, providing a localizing tool for associated brainstem dysfunction. Furthermore, abnormal vestibular potentials are related to disease severity and stage and might respect clinical and pathological asymmetry. However, further studies are warranted to reproduce the correlation of VEMP responses to the individual motor and NMSs of PD and their value as potential biomarkers at different stages of the disease, especially the early PD.

## Ethics Statement

The ethical committee of the Faculty of Medicine, Ain Shams University has allowed doing this study. The authors assert that all procedures contributing to this work comply with the ethical standards of the relevant national and institutional committees on human experimentation and with the Helsinki Declaration of 1975, as revised in 2008.

## Author Contributions

AS and DH: research idea and conception, data acquisition, data analysis and interpretation, and manuscript writing and reviewing. HE, EM-H, and JS-P: reviewing the manuscript. MS: data analysis and manuscript reviewing. OA-C: data output analysis and manuscript writing and reviewing.

## Conflict of Interest Statement

The authors declare that the research was conducted in the absence of any commercial or financial relationships that could be construed as a potential conflict of interest.
